# Emission spectra profiling of fluorescent proteins in living plant cells

**DOI:** 10.1186/1746-4811-9-10

**Published:** 2013-04-03

**Authors:** Evelien Mylle, Mirela-Corina Codreanu, Joanna Boruc, Eugenia Russinova

**Affiliations:** 1 Department of Plant Systems Biology, VIB, Technologiepark 927, Gent, 9052, Belgium; 2 Department of Plant Biotechnology and Bioinformatics, Ghent University, Technologiepark 927, Ghent, 9052, Belgium

**Keywords:** Fluorescent proteins, Gateway vectors, Spectral unmixing, Emission spectra, *Arabidopsis*

## Abstract

**Background:**

Fluorescence imaging at high spectral resolution allows the simultaneous recording of multiple fluorophores without switching optical filters, which is especially useful for time-lapse analysis of living cells. The collected emission spectra can be used to distinguish fluorophores by a computation analysis called linear unmixing. The availability of accurate reference spectra for different fluorophores is crucial for this type of analysis. The reference spectra used by plant cell biologists are in most cases derived from the analysis of fluorescent proteins in solution or produced in animal cells, although these spectra are influenced by both the cellular environment and the components of the optical system. For instance, plant cells contain various autofluorescent compounds, such as cell wall polymers and chlorophyll, that affect the spectral detection of some fluorophores. Therefore, it is important to acquire both reference and experimental spectra under the same biological conditions and through the same imaging systems.

**Results:**

Entry clones (pENTR) of fluorescent proteins (FPs) were constructed in order to create C- or N-terminal protein fusions with the MultiSite Gateway recombination technology. The emission spectra for eight FPs, fused C-terminally to the A- or B-type cyclin dependent kinases (CDKA;1 and CDKB1;1) and transiently expressed in epidermal cells of tobacco (*Nicotiana benthamiana*), were determined by using the Olympus FluoView™ FV1000 Confocal Laser Scanning Microscope. These experimental spectra were then used in unmixing experiments in order to separate the emission of fluorophores with overlapping spectral properties in living plant cells.

**Conclusions:**

Spectral imaging and linear unmixing have a great potential for efficient multicolor detection in living plant cells. The emission spectra for eight of the most commonly used FPs were obtained in epidermal cells of tobacco leaves and used in unmixing experiments. The generated set of FP Gateway entry vectors represents a valuable resource for plant cell biologists.

## Background

The completion of *Arabidopsis* and rice sequencing projects revealed many open reading frames encoding novel proteins of unknown function [1,2]. One of the major challenges for plant biologists is to allocate functions to each of these proteins by determining *in vivo* their subcellular localization and dynamics [3,4] and their complex regulatory networks of protein-protein interactions [5,6]. The availability of the genetic code of FPs and their spectral variants [7] render them as the most commonly used protein localization tools [8]. *In vivo* fluorescent labeling of virtually any protein is now possible by tagging a respective protein with a FP variant using simple molecular cloning methods and subsequent expression of the gene fusion in living cells. However, the number of proteins that can be imaged simultaneously using different FPs is still limited, not only due to the suboptimal spectroscopic and biophysical properties of some FP variants, but also their overlapping emission spectra. For these reasons, some most commonly used FPs, such as the enhanced versions of Green Fluorescent Protein (eGFP), Yellow Fluorescent Protein (eYFP), Cyan Fluorescent Protein (eCFP) or monomeric Red Fluorescent Protein (mRFP) are difficult to separate in co-localization experiments using optical filtering methods [9]. Spectral imaging expands the existing range of fluorescent microscopy applications with the possibility to simultaneously detect several distinct fluorophores with overlapping emission spectra without switching optical filters, which is essential for characterizing the proteins in their natural environment [8,10,11]. This method offers advantages in fast multicolor time-lapse measurements and advanced techniques, such as the Förster resonance energy transfer (FRET) imaging in living cells. In addition, spectral analysis is a useful tool for discriminating a true signal from autofluorescence, which is especially important for plant cell biology, as plant cells often contain pigments (e.g. polyphenols, chlorophyll) of which the emission spectra interfere with the most commonly used green or red FPs and their spectral variants [8,12,13]. The spectral imaging tool can be used to measure the emission of a single dye using a narrow emission window, which then can be compared with a single reference spectrum. Furthermore, it is useful to separate the emission spectra of different dyes obtained in parallel detection channels by linear spectral unmixing [14]. This computational technique is based on the assumption that the total detected signal for every channel can be expressed as a linear combination of the contributing fluorophores. By using simple linear equations, the signals of component fluorophores in each pixel can be “unmixed” allowing a clear separation of fluorophores with highly overlapping emission spectra. For both spectral imaging and spectral unmixing, the relative contribution of each fluorophore needs to be available as reference spectra. It is of critical importance to use accurate reference spectra, as in general emission spectra are affected not only by the components of the optical system (e.g. light source, lens, objective) but also by the experimental environment (e.g. cell and tissue types, temperature, pH) [10,11,14,15]. Therefore, the same conditions should be used for acquiring the reference and the experimental emission spectra.

In this work, we generated series of entry clones (pENTR) of several FPs in order to create C- or N-terminal protein fusions with the MultiSite Gateway recombination technology. Then, we determined the emission spectra for eight commonly used fluorophores that are fused C-terminally to either cell cycle protein cyclin-dependent kinase A;1 (CDKA;1) or CDKB1;1 [5,6] and transiently expressed in nuclei of tobacco epidermal cells. The emission spectra acquired using the Olympus FluoView™ FV1000 Confocal Laser Scanning Microscope were then compared with publicly available reference emission spectra and used for linear unmixing experiments in living plant cells. Discrepancies between the experimentally determined and publicly available emission spectra, probably due to microscope performance, were observed only for the red-shifted fluorophores.

## Results and discussion

### Generation of Gateway FP entry clones

The Gateway cloning system is based on a site-specific recombination that allows the fusion of one or more fragments in a predefined order, orientation and reading frame [16], and is commercialized by Invitrogen. To be able to use this cloning system, we created a set of 20 Gateway entry clones carrying the latest versions of five FPs: Dendra [17], Venus [18], mCherry [19], TagRFP [20] and Cerulean [21] (Table 1). Open reading frames (ORFs) of different FPs with or without a stop codon were introduced by a BP reaction into different Gateway compatible destination vectors to allow fusion with a protein of interest at its amino (N) or carboxyl (C) terminus. The FP ORFs without stop codons were introduced in pDONR™P4-P1R (Invitrogen) to create the pEN-L4-FP-R1 vectors allowing N-terminal fusion and expression under the control of the cauliflower mosaic virus (CaMV) 35S promoter [22] by using the vector pK7m24GW2 (http://gateway.psb.ugent.be/). The FP ORFs with and without stop codons were introduced in the pDONR™221 vector (Invitrogen) to create entry clones named pEN-L1-FP-L2 and pEN-L1-FP*-L2 respectively (* - stop codon). Such entry clones can be used to create N- or C-terminal fusions with promoters, genes or different epitope tags using the vector pK7m34GW (http://gateway.psb.ugent.be/). The FP ORFs with stop codons were introduced in pDONR™P2R-P3 (Invitrogen) to create pEN-R2-FP*-L3 vectors for subsequent C-terminal fusions using the vector pK7m34GW (http://gateway.psb.ugent.be/).

**Table 1:** Multi-color Gateway-compatible entry clones

**Fluorescent proteins**	**Recipient pDONR**	**Entry clone**	**att sites**
Dendra	pDONR P4-P1R	pEN-L4-Dendra-R1	attB4-attB1
	pDONR221	pEN-L1-Dendra-L2	attB1-attB2
	pDONR221	pEN-L1-Dendra*-L2	attB1-attB2
	pDONR P2R-P3	pEN-R2-Dendra*-L3	attB2-attB3
Venus	pDONR P4-P1R	pEN-L4-Venus-R1	attB1-attB2
	pDONR221	pEN-L1- Venus -L2	attB1-attB2
	pDONR221	pEN-L1- Venus *-L2	attB1-attB2
	pDONR P2R-P3	pEN-R2- Venus *-L3	attB2-attB3
mCherry	pDONR P4-P1R	pEN-L4-mCherry-R1	attB4-attB1
	pDONR221	pEN-L1- mCherry -L2	attB1-attB2
	pDONR221	pEN-L1- mCherry *-L2	attB1-attB2
	pDONR P2R-P3	pEN-R2- mCherry *-L3	attB2-attB3
TagRFP	pDONR P4-P1R	pEN-L4-TagRFP-R1	attB4-attB1
	pDONR221	pEN-L1- TagRFP -L2	attB1-attB2
	pDONR221	pEN-L1- TagRFP *-L2	attB1-attB2
	pDONR P2R-P3	pEN-R2- TagRFP *-L3	attB2-attB3
Cerulean	pDONR P4-P1R	pEN-L4-Cerulean-R1	attB4-attB1
	pDONR221	pEN-L1- Cerulean -L2	attB1-attB2
	pDONR221	pEN-L1- Cerulean *-L2	attB1-attB2
	pDONR P2R-P3	pEN-R2- Cerulean *-L3	attB2-attB3

* indicates STOP codons.

### Expression of FPs in tobacco leaf epidermal cells

To demonstrate the use of the Gateway-compatible entry clones containing different FP variants for subcellular localization of proteins in living plant cells, we created C-terminal FP fusions of the *Arabidopsis* CDKA;1 or CDKB1;1 [5,6]. Therefore, the ORF of *CDKA;1* was fused in frame to Venus [18], mCherry [19], TagRFP [20], Cerulean [21], eGFP [23-25], eYFP [23,25,26] and mRFP [23,27] whereas the ORF of *CDKB1;1* was fused in frame to eCFP [23,25]. All fusion proteins were transiently expressed under the control of the CaMV 35S promoter in tobacco (*Nicotiana benthamiana*) leaf epidermal cells. CDKA;1- and CDKB1;1-FPs were detected in the nucleus and in the cytoplasm as previously reported [6]. For control experiments, free mRFP was transiently expressed under the control of the CaMV 35S promoter in tobacco leaves.

### Emission spectra analysis

**Figure 1:**
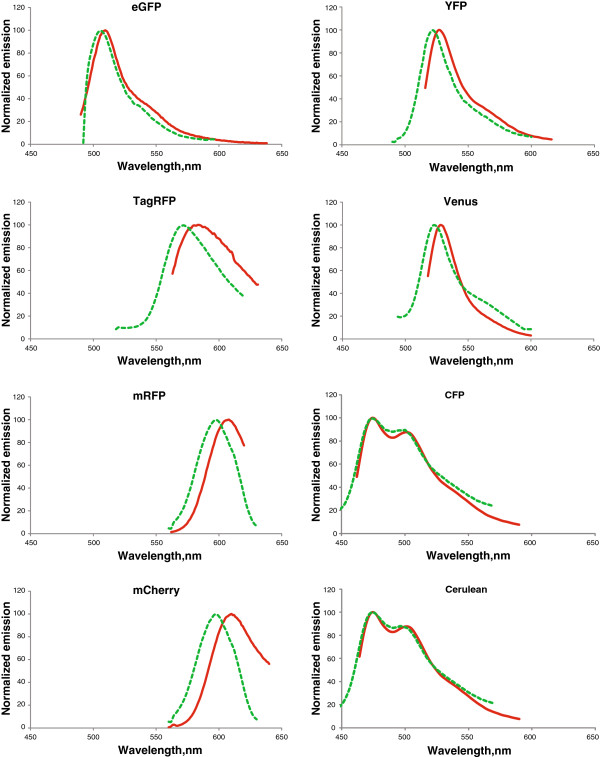
**Fluorescence emission spectra of different FPs in tobacco epidermal cells.** Eight FPs were transiently expressed in tobacco epidermal cells, and the measured fluorescence emission spectra data (green lines) were compared with the publicly available ones (red lines).

**Figure 2:**
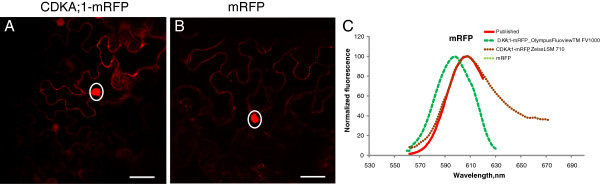
**The fluorescent emission spectra are not affected by the protein fusion.** mRFP fused to CDKA;1 (**A**) and free mRFP (**B**) were transiently expressed in tobacco and the fluorescence emission spectra were measured in the nucleus (marked by a ring) with Olympus FluoView™ FV1000 and Zeiss LSM 710 (**C**). Scale bars, 30 μm.

Multiple labeling fluorescence techniques are powerful tools for simultaneous identification of several molecular or structural components in the cell. Those techniques rely on the ability to distinguish unambiguously a number of FPs displaying overlapping spectra through linear unmixing combined with spectral imaging [14], requiring accurate reference spectra of the FPs. The spectral properties of FPs are mostly characterized in aqueous solutions [28] which cannot readily be applied to plant cells. In order to obtain the emission spectra of nine of the most commonly used FPs in living plant cells, we performed a lambda scan for each FP, fused to CDKA;1 and expressed in tobacco epidermal cells, using the Olympus FluoView™ FV1000 confocal microscope. To exclude the interference of different cellular compartments, only the nuclear pool of CDKA;1- or CDKB1; 1-FP fusions was analyzed. The excitation wavelengths were selected according to the available lasers and as close as possible to the optimal excitation wavelengths (Table 2). eCFP, eYFP, Venus and TagRFP were excited with a lower than the optimal wavelength in order to obtain the complete emission spectra. Several nuclei per fluorescent sample were analyzed, the emission fluorescence was normalized (divided by the maximum and multiplied by 100) and the average values were plotted (Figure 1, Table 2 and Additional files 1 and 2). Next, the FP spectra obtained in the plant cells were compared with publicly available spectra (Table 2, Additional file 1). A small spectral shift of 1-5 nm towards a shorter wavelength was observed for eGFP, eYFP, Venus, eCFP and Cerulean (Figure 1, Table 2 and Additional file 1). Notably, larger differences of around 10 nm were observed for the red-shifted fluorophores mRFP, mCherry, and TagRFP. To exclude the possibility that the observed changes in emission spectra are due to the fusion of the FPs to CDKA;1 we performed the same experiments when a FP, namely mRFP, was expressed as a free protein. Similar results were obtained (Figure 2A-C; Table 2; Additional file 1), suggesting that the emission spectra obtained by us are influenced by either the environment of the plant cells or the performance of the microscope. Therefore, we determined the emission spectra of mRFP expressed as a fusion with CDKA;1 in tobacco epidermal cells by using the Zeiss LSM 710 confocal microscope. In this case only a small shift of 1-2 nm was observed (Figure 2C; Table 2; Additional file 1), indicating that when measured in the nucleus of the plant cell, the emission spectra are mainly influenced by the optical system of the microscope.

**Table 2:** Characterization of different FP variants

**Fluorescent protein**	**Excitation maximum (nm)**	**Excitation wave length (nm)**	**Emission maximum according to references (nm)**	**Emission maximum measured in this study (nm)**	**Standard deviation (SD)**	**Number of nuclei analyzed (n)**	**References**
eGFP	488	488	509.5	506	1.21	20	[25]
eYFP	514	488	527	522	1	4	[25]
Venus	515	488	528	524	1.04	14	[18]
eCFP	434	405	475; 501	474; 500	1.82; 3.03	16	[25]
Cerulean	433	405	475; 501	474; 496	0.57; 2.67	12	[21]
mCherry	587	559	610	598	1.26	16	[19]
mRFP	584	559 and 561**	608	598 and 596* and 607**	1.17 and 2.19* and 1.13**	33 and 22* and 13**	[26]
TagRFP	555	515	583	572	1.56	9	[20]

* indicates experiments performed with free mRFP.

** indicates experiments performed with Zeiss LSM 710.

### Spectral fluorescence unmixing in living plant cells

**Figure 3:**
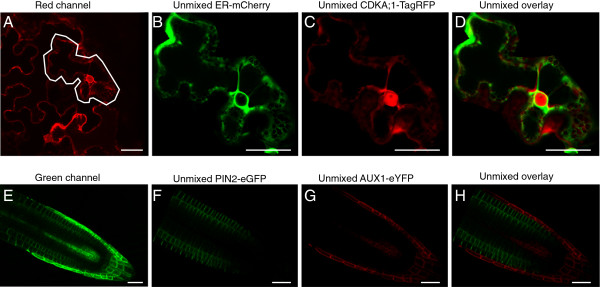
**Linear unmixing of spectrally similar fluorophores in tobacco and *Arabidopsis *cells.** (**A**-**D**) Tobacco epidermal cells transiently expressing CDKA;1-TagRFP and ER-mCherry marker. (**A**) Image before unmixing (red channel, 609-619 nm). Fluorophore signals after unmixing: ER-mCherry in the ER strands and nuclear envelope (**B**) and CDKA;1-TagRFP in the nucleus and the cytoplasm (**C**). (**D**) Overlay. (**E**-**H**) *Arabidopsis* roots expressing PIN2-eGFP and AUX1-eYFP. Image before unmixing (green channel, 524-534 nm) (**E**) and fluorophore signals after unmixing: PIN2-eGFP (**G**), AUX1-eYFP (**F**) and overlay (**H**). Scale bars, 30 μm.

A major concern for the multicolor fluorescent detection is the crossover in emission spectra between different fluorophores. Liner unmixing allows the reliable separation of overlapping fluorescent signals and subsequent accurate co-localization analysis. We next applied the linear unmixing to tobacco leaf epidermal cells or *Arabidopsis thaliana* root cells transiently or stably co-expressing two fluorophores with overlaying emission spectra. As reference spectra we used the spectra previously generated in this study (Additional file 1). We first transiently co-expressed the CDKA;1 protein fused to TagRFP and the endoplasmic reticulum (ER) marker HDEL fused to mCherry [29]. Imaging those combinations of FPs in one channel does not allow one to distinguish between mCherry and TagRFP (Figure 3A). In contrast, after acquiring a lambda stack, linear unmixing allowed the separation between the two fluorophores localized to ER strands and the nuclear envelope (ER-mCherry) [29] and to the nucleus and the cytoplasm (CDKA;1-TagRFP) [5,6] (Figure 3B-D, Additional file 3). We next used a similar approach to simultaneously analyze the localization of two proteins in *Arabidopsis* roots, namely the auxin influx and efflux carrier components, PIN2 and AUX1, fused to eGFP and eYFP, respectively [30,31] (Figure 3E-H). As previously shown [30,31], after linear unmixing, the localization of PIN2 was detected in the cortical and epidermal cells of the root (Figure 3F and H), whereas AUX1 was localized in a subset of columella, lateral root cap and stele tissues (Figure 3G and H).

## Conclusions

The localization of proteins in living cells is facilitated by the availability of many FP variants with different spectral proprieties [28], allowing the simultaneous *in vivo* visualization of different proteins, elucidating their subcellular localization, trafficking from one compartment to another, and possible protein-protein interactions. However, non-overlapping fluorescence excitation/emission spectra are required for these kind of analyses. Currently, the excitation/emission maxima for FPs are identified using pure proteins in aqueous solutions. However, in plant systems, the cellular environment could be different, depending on the developmental stage, tissue or cell type, which can influence the spectral outcome. Therefore, we analyzed the emission spectra of nine FPs *in vivo* in tobacco leaves. We found that FPs measured in plant cells have emission curves similar to those published by others [28], and more influenced by the performance of the microscope than by the nuclear environment of the plant cell. However, we cannot exclude the possibility that other locations in the plant cell might influence the function of the fluorophores more substantially. Next, we demonstrated that the emission spectra obtained by us can be used to perform linear unmixing experiments in living plant cells.

In addition, the generated collection of 20 Gateway entry clones carrying different FPs is a valuable resource for the plant research community and can be applied for a variety of analyses: promoter activity, gene expression, imaging intracellular molecular dynamics and protein-protein interactions. All vectors described here are documented with maps, Vector NTI, and sequence files, and can be requested online at http://gateway.psb.ugent.be/.

## Methods

### Vector construction

The full-length ORFs of Dendra, Venus, mCherry, TagRFP and Cerulean FPs were amplified by PCR using the following templates: Gateway Dendra2-At-C entry clone (Evrogen), VAN3-Venus [32], mCherry (pER-rk CD3-959) [29], TagRFP (Evrogen) and Cerulean (Clontech). Oligonucleotide primers used in PCR reactions to generate att sites and for sequencing are listed in Additional file 4. The PCR products were then introduced into different entry clones: pDONR™P4-P1R, pDONR™221 or pDONR™P2R-P3 (Invitrogen) in order to generate 20 vectors containing specific FPs (Table 1). All inserts were fully sequenced to verify that no PCR or cloning errors occurred. Details of the entry clones containing different FPs can be found on the Web site (http://gateway.psb.ugent.be/). The Venus, mCherry, TagRFP and Cerulean FPs in pDONR™P2R-P3 were recombined with the destination vectors pK7m34GW, the *CDKA;1* gene in pDONR™221 and the CaMV 35S promoter in pDONR™P4-P1R. *CDKA;1* gene in pDONR™221 was introduced into the destination vectors, pK7FWG2 (containing eGFP), pH7YWG2 (containing eYFP) and pH7RWG2 (containing mRFP), whereas *CDKB1;1* gene in pDONR™221 was introduced into the pK7CWG2 (containing eCFP). pK7FWG2, pH7YWG2, pH7RWG2 and pK7CWG2 vectors were already available in our Gateway collection (http://gateway.psb.ugent.be/) [33-35]. The free mRFP construct was created by introducing mRFP in pDONR™221 into the destination vector pK7WG2 (http://gateway.psb.ugent.be/). All constructs in the destination vectors were sequenced to verify that correct fragments were cloned in frame. Plasmid extractions for routine DNA manipulation and sequencing were done using miniprep purification kit Nucleobond (Clontech Inc. Lab) according to the manufacturer’s protocol.

### Expression analysis and plant material

Constructs containing different FPs were introduced into *A. tumefaciens*, and then infiltrated into wild-type tobacco (*Nicotiana benthamiana*) plants, according to the method previously described [6]. The nuclear localization was analyzed 3 to 5 days after infiltration. The PIN2-GFP and AUX1-YFP *Arabidopsis*-expressing lines were described previously [30,31]. *Arabidopsis* seedlings were stratified for 2 days at 4°C and germinated on vertical agar plates with half-strength Murashige and Skoog (½ MS) with 1% (w/v) sucrose at 22°C in a 16 h-8 h light–dark cycle for 4 days before imaging.

### Confocal microscopy, emission spectra analysis and linear unmixing

Lambda stacks for each fluorophore were acquired with a confocal microscope Olympus FluoView™ FV1000 (Tokyo, Japan), with a 63× water corrected objective (numerical aperture of 1.2). The emission light was captured using a bandwidth of 10 nm, with a 2 nm step. The saturation level was verified for each image. The start and end wavelength were chosen in function of the fluorophore: 5 nm after the excitation wavelength, always an odd or even number, and ending on a wavelength that is around 30 nm away from the emission peak. After taking the lambda stack, the original settings were used to capture an image of the fluorophore again, in order to make sure that the cell remained in-focus. The following dichroic mirrors were selected as a function of the respective spectra for imaging with the Olympus FluoView™ FV1000, BS20/80 (Cerulean and CFP), BS405/488 (eYFP, eGFP and Venus), BS408/488/559/635 (mCherry and mRFP) and BS458/515 (TagRFP). Dichroic mirror BS488/561 was used for imaging of mRFP with the Zeiss LSM 710. For each FP the fluorescence emission spectra were recorded in a λ-spectral mode as presented in Additional file 1. The data were analyzed with the ‘Series analysis’ tool of the Olympus FluoView™ FV1000 software. Whole nuclei were selected as regions of interest (ROIs) and average intensity values were exported to Excel, normalized (the respective value was divided by the maximum and multiplied by 100), averaged and plotted. For unmixing of different fluorophores in one sample, a lambda stack was performed on similar way as described above. To unmix the fluorophores, the software tool ‘Spectral unmixing’ of the Olympus FluoView™ was used with the spectra of the fluorophore alone as a reference and activating the background correction. Zeiss LSM 710 was also used for obtaining the fluorescence emission spectra of mRFP. A band width of 3 nm and a range from 562 to 700 were selected. The unmixing tool of the ZEN software was used to obtain the emission spectra of each selected nuclei. Intensity values were exported to Excel, normalized, averaged and plotted.

## Competing interests

The authors declare that they have no competing interests.

## Authors’ contributions

EM conducted the lambda scans and performed the linear unmixing experiments. M-CC created the entry clones containing FPs. EM and JB created the expression vectors containing FPs. M-CC, EM and JB expressed the vectors in tobacco epidermis. EM, M-CC, JB and ER designed the experiments, analyzed the data. ER wrote the manuscript. All authors read and approved the final manuscript.

## Supplementary Material

Additional file 1 Fluorescence emission spectra of different FPs. Click here for file

Additional file 2 **Example of fluorescence emission spectra analysis.** Venus fused to CDKA;1 was transiently expressed in tobacco (A). 16 nuclei were analysed as shown in (B). The emission fluorescence was normalized (divided by the maximum and multiplied by 100) (C) and the average value was plotted (D). Scale bar, 30 μm in (A) and 2 μm in (B). Click here for file

Additional file 3 **Control expression analysis.** Expression of single fluorescently tagged proteins. Tobacco epidermal cells transiently expressing CDKA;1-TagRFP (A) and ER-mCherry marker (B). Scale bars, 30 μm. Click here for file

Additional file 4 List of primers used for cloning of the FPs. Click here for file
