# Supplementary figures and images for: Emission spectra profiling of fluorescent proteins in living plant cells
Source: Plant Methods. 2013 Apr 3;9:10. doi: 10.1186/1746-4811-9-10 (PMC3630006; doi:10.1186/1746-4811-9-10)

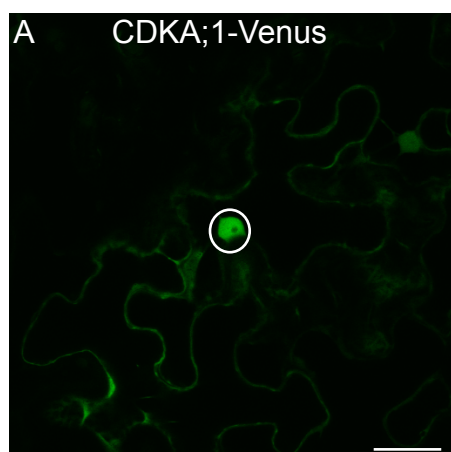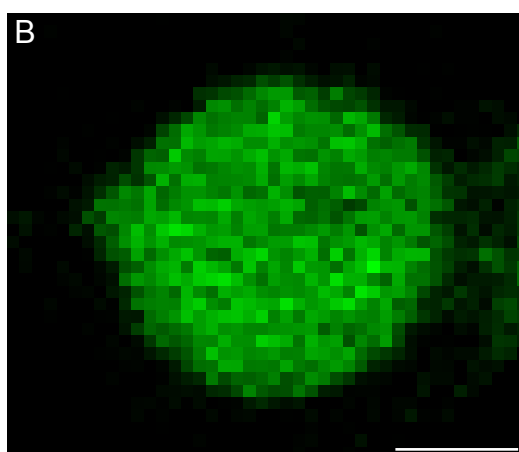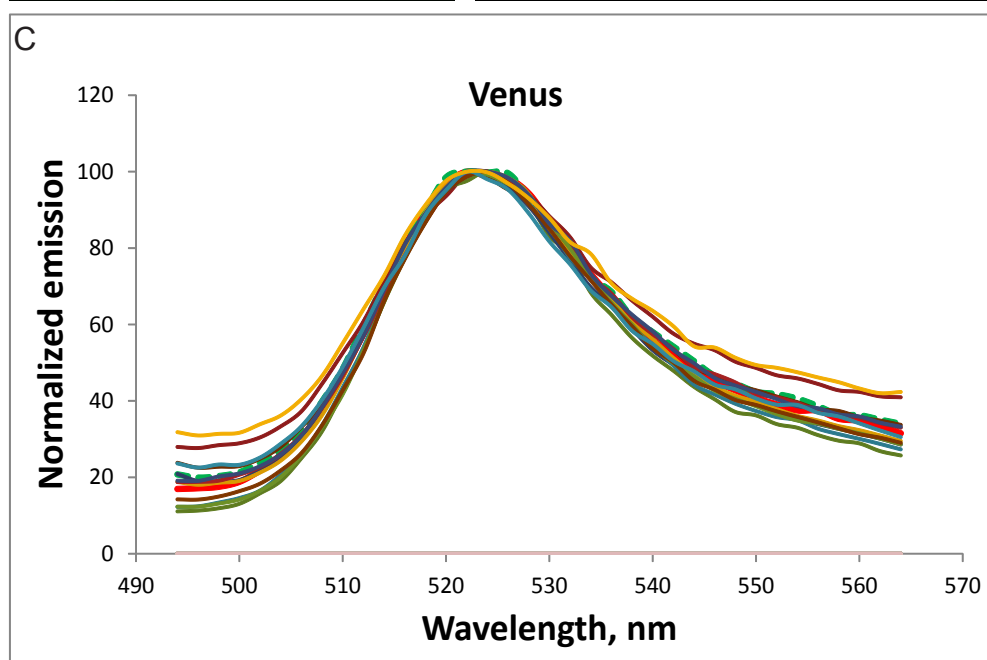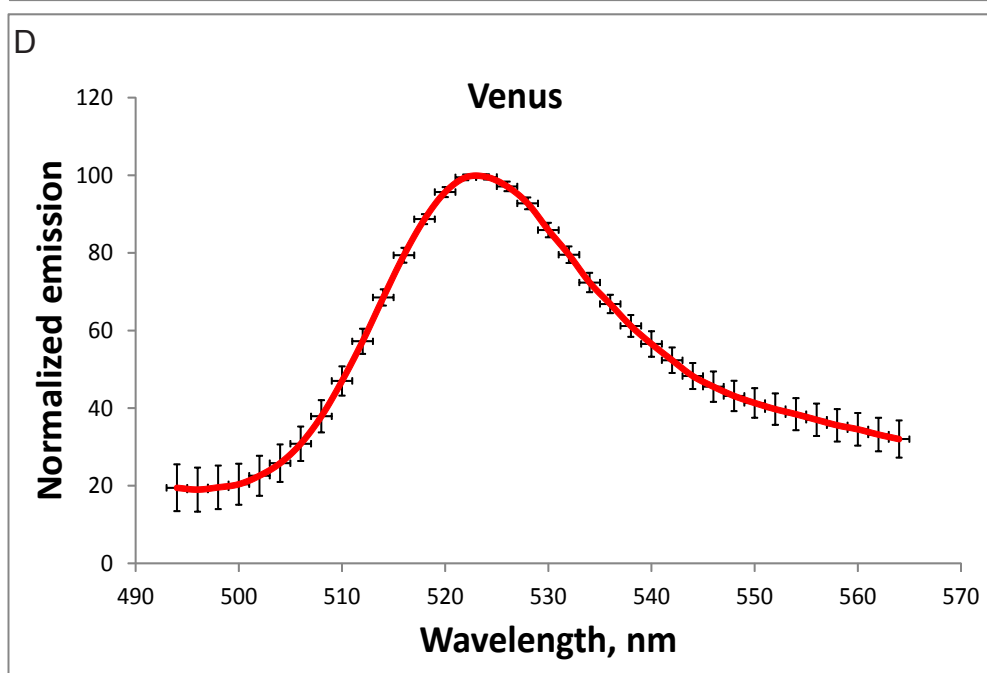

Supplement: Additional file 2 — Example of fluorescence emission spectra analysis. Venus fused to CDKA;1 was transiently expressed in tobacco (A). 16 nuclei were analysed as shown in (B). The emission fluorescence was normalized (divided by the maximum and multiplied by 100) (C) and the average value was plotted (D). Scale bar, 30 μm in (A) and 2 μm in (B). [file 1746-4811-9-10-S2.pdf]

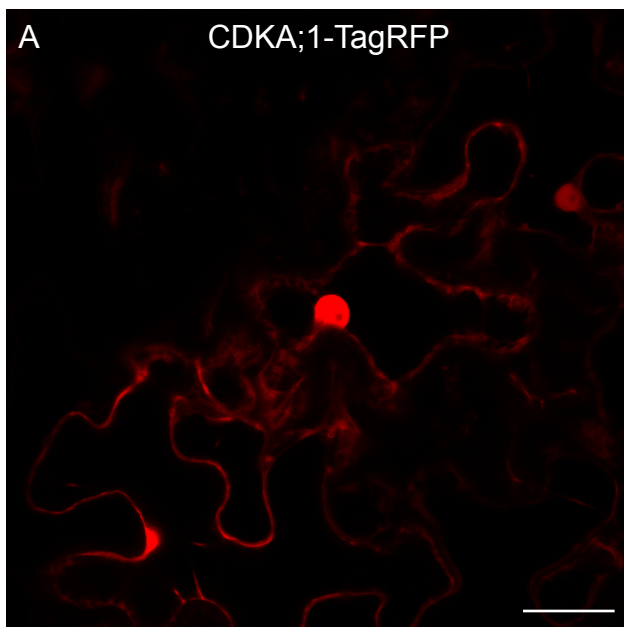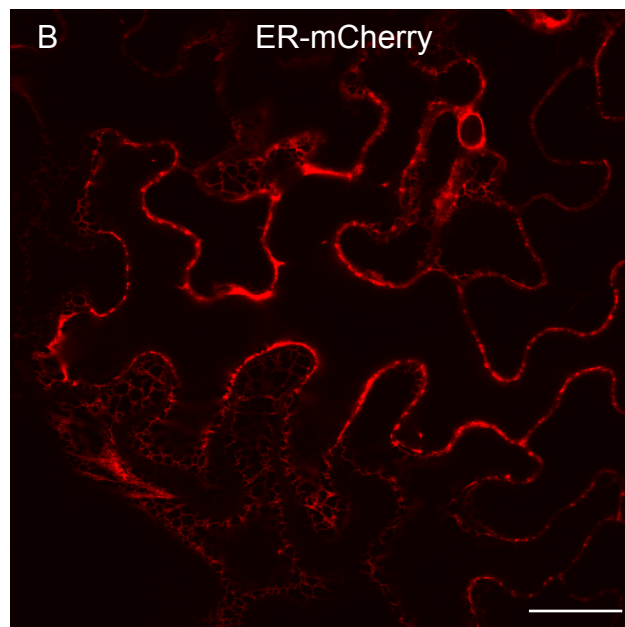

Supplement: Additional file 3 — Control expression analysis. Expression of single fluorescently tagged proteins. Tobacco epidermal cells transiently expressing CDKA;1-TagRFP (A) and ER-mCherry marker (B). Scale bars, 30 μm. [file 1746-4811-9-10-S3.pdf]
