# Supplementary material for: Emission spectra profiling of fluorescent proteins in living plant cells
Source: Plant Methods. 2013 Apr 3;9:10. doi: 10.1186/1746-4811-9-10 (PMC3630006; doi:10.1186/1746-4811-9-10)
Supplement: Additional file 4 — List of primers used for cloning of the FPs. [file 1746-4811-9-10-S4.pdf]

Additional file 4. List of primers used for cloning of the FPs

	Primer	att sites	Sequence
1	Dendra_4-1_ F	attB4	GGGGACAACTTTGTATAGAAAAGTTGCT **ATGAACACTCCTGGAATC**
2	Dendra _4-1_ R	attB1	GGGGACTGCTTTTTTGTACAAACTTGC **CCAAACCTGTGATGGGAG**
3	Dendra _1-2_ F	attB1	GGGGACAAGTTTGTACAAAAAAGCAGGCTTA **ATGAACACTCCTGGAATC**
4	Dendra _1-2_ R	attB2	GGGGACCACTTTGTACAAGAAAGCTGGGTA **CCAAACCTGTGATGGGAG**
5	Dendra *_1-2_ F	attB1	GGGGACAAGTTTGTACAAAAAAGCAGGCTTA **ATGAACACTCCTGGAATCAATCT**
6	Dendra *_1-2_ R	attB2	GGGGACCACTTTGTACAAGAAAGCTGGGTA **TTACCAAACCTGTGATGG**
7	Dendra *_2-3_ F	attB2	GGGGACAGCTTTCTTGTACAAAGTGGCT **ATGAACACTCCTGGAATC**
8	Dendra *_2-3_ R	attB3	GGGGACAACTTTGTATAATAAAGTTGC **TTACCAAACCTGTGATGG**
9	Venus_4-1_ F	attB4	GGGGACAACTTTGTATAGAAAAGTTGCT **ATGGTGAGCAAGGGCGAG**
10	Venus _4-1_ R	attB1	GGGGACTGCTTTTTTGTACAAACTTGC **CTTGTACAGCTCGTCCAT**
11	Venus _1-2_ F	attB1	GGGGACAAGTTTGTACAAAAAAGCAGGCTTA **ATGGTGAGCAAGGGCGAG**
12	Venus _1-2_ R	attB2	GGGGACCACTTTGTACAAGAAAGCTGGGTA **CTTGTACAGCTCGTCCATGCC**
13	Venus *_1-2_ F	attB1	GGGGACAAGTTTGTACAAAAAAGCAGGCTTA **ATGGTGAGCAAGGGCGAG**
14	Venus *_1-2_ R	attB2	GGGGACCACTTTGTACAAGAAAGCTGGGTA **TTACTTGTACAGCTCGTCCATGCC**
15	Venus *_2-3_ F	attB2	GGGGACAGCTTTCTTGTACAAAGTGGCT **ATGGTGAGCAAGGGCGAG**
16	Venus *_2-3_ R	attB3	GGGGACAACTTTGTATAATAAAGTTGC **TTACTTGTACAGCTCGTCCATGCCG**
17	mCherry_4-1_ F	attB4	GGGGACAACTTTGTATAGAAAAGTTGCT **ATGGTGAGCAAGGGCGAG**
18	mCherry _4-1_ R	attB1	GGGGACTGCTTTTTTGTACAAACTTGC **CTTGTACAGCTCCTCCAT**
19	mCherry _1-2_ F	attB1	GGGGACAAGTTTGTACAAAAAAGCAGGCTTA **ATGGTGAGCAAGGGCGAG**
20	mCherry _1-2_ R	attB2	GGGGACCACTTTGTACAAGAAAGCTGGGTA **CTTGTACAGCTCCTCCATGCC**
21	mCherry *_1-2_ F	attB1	GGGGACAAGTTTGTACAAAAAAGCAGGCTTA **ATGGTGAGCAAGGGCGAG**
22	mCherry *_1-2_ R	attB2	GGGGACCACTTTGTACAAGAAAGCTGGGTA **TTACTTGTACAGCTCCTCCATGCC**
23	mCherry *_2-3_ F	attB2	GGGGACAGCTTTCTTGTACAAAGTGGCT **ATGGTGAGCAAGGGCGAG**
24	mCherry *_2-3_ R	attB3	GGGGACAACTTTGTATAATAAAGTTGC **TTACTTGTACAGCTCCTCCATGCCG**
25	TagRFP_4-1_ F	attB4	GGGGACAACTTTGTATAGAAAAGTTGCT **ATGGTGTCTAAGGGCGAA**
26	TagRFP _4-1_ R	attB1	GGGGACTGCTTTTTTGTACAAACTTGC **ATTAAGTTTGTGCCCCAGTTT**
27	TagRFP _1-2_ F	attB1	GGGGACAAGTTTGTACAAAAAAGCAGGCTTA **ATGGTGTCTAAGGGCGAA**
28	TagRFP _1-2_ R	attB2	GGGGACCACTTTGTACAAGAAAGCTGGGTA **ATTAAGTTTGTGCCCCAG**
29	TagRFP *_1-2_ F	attB1	GGGGACAAGTTTGTACAAAAAAGCAGGCTTA **ATGGTGTCTAAGGGCGAA**

30	TagRFP *_1-2_ R	attB2	GGGGACCACTTTGTACAAGAAAGCTGGGTA **TCAATTAAGTTTGTGCCC**
31	TagRFP *_2-3_ F	attB2	GGGGACAGCTTTCTTGTACAAAGTGGCT **ATGGTGTCTAAGGGCGAA**
32	TagRFP *_2-3_ R	attB3	GGGGACAACTTTGTATAATAAAGTTGC **TCAATTAAGTTTGTGCCC**
33	Cerulean_4-1_ F	attB4	GGGGACAACTTTGTATAGAAAAGTTGCT **ATGGTGAGCAAGGGCGAG**
34	Cerulean _4-1_ R	attB1	GGGGACTGCTTTTTTGTACAAACTTGC **ACTGGGTGCTCAGGTAGT**
35	Cerulean _1-2_ F	attB1	GGGGACAAGTTTGTACAAAAAAGCAGGCTTA **ATGGTGAGCAAGGGCGAG**
36	Cerulean _1-2_ R	attB2	GGGGACCACTTTGTACAAGAAAGCTGGGTA **ACTGGGTGCTCAGGTAGT**
37	Cerulean *_1-2_ F	attB1	GGGGACAAGTTTGTACAAAAAAGCAGGCTTA **ATGGTGAGCAAGGGCGAG**
38	Cerulean *_1-2_ R	attB2	GGGGACCACTTTGTACAAGAAAGCTGGGTA **TTAACTGGGTGCTCAGGT**
39	Cerulean *_2-3_ F	attB2	GGGGACAGCTTTCTTGTACAAAGTGGCT **ATGGTGAGCAAGGGCGAG**
40	Cerulean *_2-3_ R	attB3	GGGGACAACTTTGTATAATAAAGTTGC **TTAACTGGGTGCTCAGGT**
*- indicates STOP codon			
